# Visualizing *in situ* viral replication across the natural history of chronic HBV infection

**DOI:** 10.1097/HC9.0000000000000111

**Published:** 2023-03-30

**Authors:** Hanyue Zhang, Miaoqu Zhang, Qiran Zhang, Yiqi Yu, Fan Zhang, Jing Wang, Mingzhe Zhou, Tong Yu, Chuan Shen, Shuili Yu, Yanfang Huang, Yuxian Huang, Jiming Zhang, Jie Jin, Chao Qiu, Li Guojun, Wenhong Zhang

**Affiliations:** 1 Department of Infectious Disease, National Medical Center for Infectious Diseases and Shanghai Key Laboratory of Infectious Diseases and Biosafety Emergency Response, Huashan Hospital, Fudan University, Shanghai, P.R. China; 2 Department of Gastroenterology, Sir Run Run Shaw Hospital, Zhejiang University School of Medicine, Hangzhou, Zhejiang Province, P.R. China; 3 Department of Liver Disease, The Third People’s Hospital of Shenzhen, Shenzhen, Guangdong Province, P.R. China; 4 Department of Infectious Disease, Jing'an District Central Hospital, Shanghai, China.; 5 Clinical Research Center for Infectious Disease of Hebei Province, Shijiazhuang, Hebei Province, P.R. China; 6 The Second Hospital of Yinzhou of Ningbo, Ningbo, P.R. China; 7 Key Laboratory of Medical Molecular Virology (MOE/MOH) and Institutes of Biomedical Sciences, Fudan University, Shanghai, P.R. China

## Abstract

Chronic HBV infection evolves through different phases. Interactions between viral replication and the host immune response in the liver underlie the pathogenesis of this disease. The aim of this study was to directly visualize the HBV replication intermediates at a single-cell resolution inscribed on morphological changes corresponding to disease activity.

**Methods:**

A set of archived formalin-fixed paraffin-embedded liver needle biopsies from treatment-naïve patients were collected and categorized into phases according to the American Association for the Study of the Liver Diseases (AASLD) guidelines. HBV RNA and DNA were detected using *in situ* hybridization assays.

**Results:**

The hepatocytes were ubiquitously infected in subjects with immune tolerance, and their percentage was gradually decreased in immune-active and inactive chronic hepatitis B phases. HBV-infected hepatocytes were prone to localize close to fibrous septa. The subcellular distribution of signals was able to distinguish hepatocytes with productive infection from those harboring HBV integrants and transcriptionally inactive covalently closed circular DNAs. A smaller number of hepatocytes with productive infection and more harboring transcriptionally inactive covalently closed circular DNA or HBV integrants became apparent in the inactive chronic hepatitis B phase.

**Conclusion:**

An atlas of *in situ* characteristics of viral-host interactions for each phase is described, which sheds light on the nature of viral replication and disease pathogenesis among the phases of chronic HBV infection.

## Graphical abstract

**Figure:**
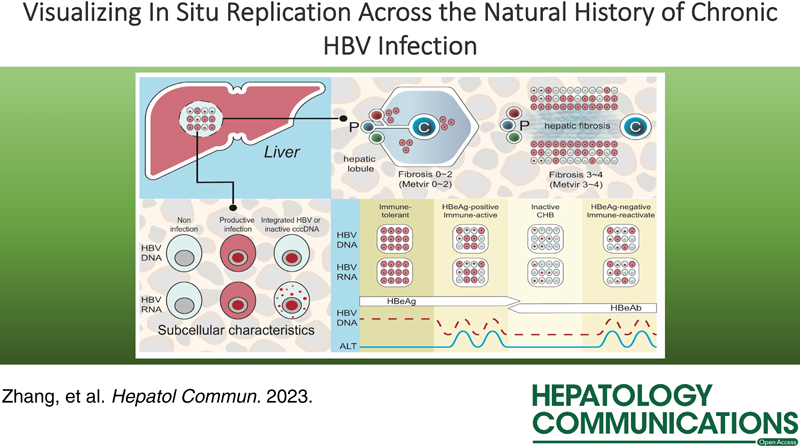

## INTRODUCTION

The natural history of chronic HBV infection typically evolves through different phases, from an early stage characterized by high HBV replication and low signs of inflammation to an immunopathogenic phase wherein virus-infected hepatocytes are progressively cleared by inadequate adaptive immune responses until it reaches a lap that spontaneously suppresses viral replication to a low level, or eventually achieves a cure in a rare number of cases.^1^–^4^ In clinical practice, these phases are usually delineated through the laboratory assessment of biomedical, serological, and virological circulating biomarkers to interpret disease manifestation in the liver, because of the convenient access in the outpatient setting. The mechanisms underlying intrahepatic viral replication and the viral-host interaction are believed to be distinct for each phase, which is a progressive course. However, serum markers cannot yield a comprehensive outlook on *in situ* disease activity.^5^–^10^ Therefore, investigating viral replicative intermediates in clinical liver specimens is encouraged to improve our understanding of the phases of natural history and pathogenesis of chronic HBV infection.

In most studies, the amounts of different viral intermediates are usually quantified using bulk nucleic acids extracted from liver specimens. These lose the spatial information offered by tissue architectures and ignore the complexity of cell-to-cell heterogeneity.^11^–^16^ In this study, we directly visualized the viral intermediate species at a single-cell resolution on liver biopsy sections using *in situ* hybridization, which also allowed us to enumerate the infected individual cells and observe the *in situ* cell state of viral replication based on observed morphological changes that correspond to disease activity. Our findings revealed that both the number of infected hepatocytes and the in-cell state of viral replication are distinct, particularly in 2 extreme phases, the immune-tolerant (IT) and inactive chronic hepatitis B (IC) phases. An atlas of *in situ* characteristics of viral-host interaction for each phase is described, which contributes to our knowledge of intrahepatic viral replication and disease pathogenesis across the course of chronic HBV infection.

## METHODS

### Patients and samples

In this study, all the patients were diagnosed with chronic HBV infection according to the American Association for the Study of the Liver Diseases (AASLD) guidelines.^3^,^4^ Patients infected with other forms of viral hepatitis, including autoimmune hepatitis, metabolic liver diseases, alcoholic-associated liver disease, as well as those with HIV coinfection and HCC, were excluded. Sixty patients with chronic HBV infection were initially enrolled, and 13 patients were excluded: 8 patients with incomplete clinical data, 2 patients possibly with drug-induced hepatitis, and 3 patients with poorly stained pathological sections for nucleic acid degradation. A total of 47 patients with chronic HBV infection were included, and their demographical and clinical information are summarized in Table 1. All the patients were categorized into phases according to the AASLD guidelines^3^,^4^: IT (n=7), HBeAg-positive immune-active (HBeAg+IA, n=16), IC (IC, n=7), and HBeAg-negative immune reactive (HBeAg−IA, n=17). To categorize patients into appropriate phases, patients with equivocal HBeAg and anti-HBeAg levels were excluded, liver inflammation was evaluated based on alanine aminotransferase levels and liver histology, and IT and HBeAg-negative patients were confirmed through re-evaluation after 3–4 months. A needle liver biopsy was collected before receiving antiviral treatment. Inflammation and fibrosis in the liver biopsy were evaluated based on the METAVIR standard. Besides, inflammation was evaluated based on the Histological Activity Index standard at the same time. The study protocol was approved by the Ethical Committee of Huashan Hospital (HE2022021) and was carried out according to the Declaration of Helsinki. Written consent was given in writing by all patients. Demographic and clinical information was collated from the patients’ medical records.

**TABLE 1:** Patient characteristics

Phase	Case no.	Sex	Age	HBeAg/anti-HBe	HBV DNA (log IU/mL)	HBsAg (log IU/mL)	ALT (IU/L)	AST (IU/L)	Metavir activity stage (/3)	Metavir fibrosis stage (/4)
Immune-tolerant	Pt.1	F	19	+/−	7.05	4.46	33	28	2	2
	Pt.2	M	33	+/−	8.23	4.81	27	22	1	1
	Pt.3	F	41	+/−	7.26	—	17	20	2	1
	Pt.4	F	42	+/−	6.44	5.10	24	22	2	2
	Pt.5	M	40	+/−	7.71	5.20	19	19	2	1
	Pt.6	M	28	+/−	7.78	4.79	19	23	0	0
	Pt.7	M	23	+/−	7.79	4.78	19	17	1	1
HBeAg (+) IA	Pt.8	F	26	+/−	6.08	3.03	62	57	3	2
	Pt.9	F	41	+/−	5.56	3.26	113	64	3	2
	Pt.10	M	32	+/−	5.32	3.78	46	35	1	4
	Pt.11	F	48	+/−	8.10	3.79	75	59	3	4
	Pt.12	M	36	+/−	8.23	4.57	301	231	3	4
	Pt.13	M	51	+/−	5.06	—	41	53	3	4
	Pt.14	M	43	+/−	4.67	—	20	29	3	3
	Pt.15	M	46	+/−	4.90	0.08	49	33	3	3
	Pt.16	M	39	+/−	4.70	3.67	33	24	2	4
	Pt.17	M	45	+/−	5.13	3.41	94	68	3	3
	Pt.18	F	40	+/−	5.51	4.49	46	45	2	1
	Pt.19	F	35	+/−	7.29	—	33	32	3	2
	Pt.20	M	28	+/−	7.12	3.86	96	63	2	2
	Pt.21	M	33	+/−	6.95	3.91	104	39	2	1
	Pt.22	M	27	+/−	7.89	3.95	394	156	1	0
	Pt.23	M	37	+/−	4.62	—	22	23	1	1
Inactive chronic hepatitis B	Pt.24	F	34	−/+	TND	3.00	16	19	1	2
	Pt.25	M	36	−/+	2.34	3.25	24	17	1	2
	Pt.26	M	31	−/+	TND	3.54	21	24	1	4
	Pt.27	M	36	−/+	2.54	1.77	112	58	1	1
	Pt.28	M	51	−/+	2.16	3.47	33	31	2	4
	Pt.29	M	52	−/+	2.61	1.73	55	40	1	2
	Pt.30	M	38	−/+	—	1.47	—	—	1	1
HBeAg (−) IA	Pt.31	M	55	−/+	4.77	2.43	245	132	3	4
	Pt.32	M	45	−/+	5.06	2.72	103	152	3	3
	Pt.33	M	30	−/+	5.30	2.82	399	110	3	3
	Pt.34	M	48	−/+	6.08	3.69	67	56	2	4
	Pt.35	M	37	−/+	4.38	3.77	37	19	2	3
	Pt.36	M	38	−/+	7.52	4.21	79	57	2	3
	Pt.37	M	56	−/+	TND	—	31	29	3	3
	Pt.38	M	43	−/+	6.04	—	39	32	3	2
	Pt.39	M	32	−/+	3.77	—	28	59	2	3
	Pt.40	M	44	−/+	5.36	3.33	55	28	2	2
	Pt.41	M	46	−/+	4.33	3.41	26	22	2	4
	Pt.42	M	41	−/+	TND	—	31	20	1	1
	Pt.43	M	40	−/+	3.50	0.91	102	130	1	4
	Pt.44	M	51	−/+	5.58	3.26	26	25	2	4
	Pt.45	M	37	−/+	TND	2.93	41	33	3	4
	Pt.46	M	56	−/+	5.09	3.59	40	34	1	2
	Pt.47	M	35	−/+	3.74	3.60	19	23	1	2

Abbreviations: ALT, alanine aminotransferase; AST, aspartate aminotransferase; F, female; IA, immune-active; M, male; —, not available; Pt., patient; TND, target not detected.

### *In situ* hybridization

*In situ* hybridization was performed on routine paraffin sections of the liver needle biopsies. The probes used in this study can cover the full length of the A–D genotype of the HBV genome, with high sensitivity for single-molecule nucleic acid detection and reliable specificity^17^ (Supplemental Figure S1, http://links.lww.com/HC9/A227). The V-HBV-536207-O4 and V-HBV-536207-04-sense probes were synthesized to hybridize with the HBV DNA plus and minus strands, respectively, by Advanced Cell Diagnostics. The experimental procedures were adapted and modified based on the manufacturer’s instruction of RNAscope2.5 HD Detection Reagent Kit-RED (No. 322360; ACDbio). Upon detecting the HBV RNA, to remove DNA, the section was predigested with RNase-free DNase I (Takara) for 30 minutes at 37°C after Protease Plus treatment, then subjected to hybridization with V-HBV-536207-O4. The HBV minus-strand was directly probed with V-HBV-536207-04-sense that would not bind to the HBV RNA. After adding the V-HBV-536207-04-sense probe, the sample was incubated at 95°C for 5 minutes to denature the dsDNA to improve the efficiency of annealing of the probe to the target. The probes in the tissue were finally visualized using the Fast Red dye substrate, and the cell nucleus was counterstained with Gill’s hematoxylin I. Sections with obscure signals were not included in further image analyses.

### Quantification of HBV DNA-positive and HBV RNA-positive signals

K-VIEWER, version 1 was used to preview the whole slice and capture images in randomly selected nonrepetitive fields under ×200 magnification. Qupath, version 0.3.0 was used to count all hepatocytes,^18^ the number of cells with positive signals, and the number of cells with a different subcellular signal distribution. The percentage of positive cells in each section was calculated by averaging the percentage of positive cells for all the specified fields from the same section. To observe the histological structure of a functional unit of the liver, 2–5 portal areas and adjacent central venous visual fields were selected, and images were captured per lobule. For patients with fibrosis stages 0, 1, and 2, the percentage of positive cells in a 100 μm area around the portal vein or the central vein was counted. For patients with fibrosis stages 3–4, the percentages of positive cells within 100 μm and 100–200 μm areas around the fibrous septa were calculated.

### Statistical analysis and image processing

Data are expressed as means and SDs, or medians and interquartile ranges. GraphPad Prism, version 8.0.2 was used to analyze the data and generate figures. Nonparametric tests, including the Mann-Whitney test, Wilcoxon matched-pairs signed-rank test, and Kruskal-Wallis 1-way ANOVA followed by the Dunn test, if appropriate, were performed using SPSS 19.0. All tests were 2-tailed, and a *p*-value <0.05 was considered statistically significant. Photoshop CS6 was used to improve the contrast between signals and noise in histological images.

## RESULTS

**FIGURE 1:**
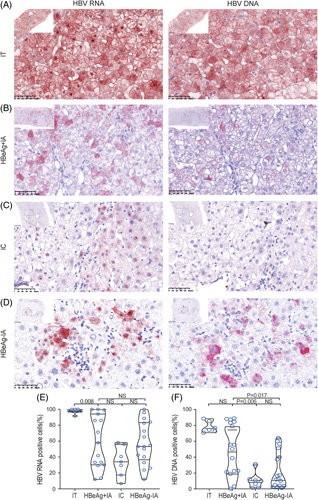
Representative images of *in situ* hybridization staining of liver sections. (A) Representative images of sections from immune-tolerant (IT) patients. The majority of the hepatocytes are positive. (B) Representative images of sections from HBeAg-positive immune-active (HBeAg+IA) patients. A proportion of hepatocytes are positive for both HBV RNA and DNA. (C) Representative images of sections from inactive chronic hepatitis B (IC) patients. Only a few hepatocytes are positive for HBV RNA and DNA, and most signals are only seen in the nucleus. (D) Representative images of sections from HBeAg-negative immune-active (HBeAg−IA) patients. A small proportion of hepatocytes are positive for HBV RNA and DNA (×400). (E) The percentage of HBV RNA-positive hepatocytes in hepatitis B patients. (F) The percentage of HBV DNA-positive hepatocytes in hepatitis B patients. Abbreviation: NS, nonsignificant.

We screened hundreds of archived formalin-fixed paraffin-embedded liver needle biopsies and detected HBV RNA and HBV DNA in connective tissue sections. A preliminary evaluation confirmed that the HBV RNA and DNA signals were strictly distributed in hepatocytes and not in other cell types, suggesting high specificity in our experimental approach. The nonspecific staining sections and those with low signal-to-noise ratios had been excluded from further analysis. HBV RNA and DNA were successfully detected on the liver needle biopsy sections from a total of 47 patients, including 7 subjects in the IT phase, 16 patients in the HBeAg+IA phase, 7 patients in the IC phase, and 17 patients in the HBeAg−IA phase (Table 1, Figure 1, Supplemental Figure S2, http://links.lww.com/HC9/A228). We microscopically observed >1300 nonrepetitive fields, including 940 hepatic lobular areas, over 200 fields around the portal triads and central vein areas, and 200 fibrous septa. In subjects in the IT phase with an HBV-productive infection, the signals were widespread across the lobules and were ubiquitous in almost every hepatocyte. In most cases in the latter 3 phases, the HBV RNA and DNA signals were scattered and irregularly distributed in the tissue.

### Frequency of HBV-infected hepatocytes across the phases of the disease

We first determined the percentage of HBV RNA-positive or DNA-positive hepatocyte. Agreeing with the serum HBV RNA and DNA load, both the percentages of HBV RNA-positive and DNA-positive cells were uniformly high in each subject in the IT phase, whereas those in the other phases had a broad range of deviation, suggesting a very dynamic intrahepatic course of viral infection transitioning from the early productive infection into the latter 3 phases. Statistically, the percentage of HBV RNA-positive cells was the highest in the IT phase [97.8% (97.2%, 100%), n=7], which was significantly higher than that in the HBeAg+IA [57.9% (31.9%, 90.1%), n=16], IC [34.0% (23.4%, 48.3%), n=7], or HBeAg−IA phases [53.2% (40.3%, 82.6%), n=17] (Figure 1B, Table 2). No significant difference was observed among the latter 3 phases, albeit a reducing trend was noted in the IC phase. The percentage of HBV DNA-positive cells was also higher in the IT [75.5% (73.0%, 83.1), n=7] and HBeAg+IA phases [52.9 (22.2%, 83.6%), n=16], compared those in the IC [9.9% (4.2%, 11.7%), n=7] and HBeAg−IA phases [12.5% (4.2%, 40.1%), n =17] (Figure 1C, Table 2). An increase in sample size may help reach a significant difference between the IT and IC phases since the results were nearly uniform in subjects in the IT phase.

**TABLE 2:** Subcellular characteristics of positive signals in each patient

		RNA-positive			DNA-positive		
Phase	Case no.	Total	Nucleus punctum	Cytoplasmic sand or punctum like	Total	Nucleus punctum	Cytoplasmic sand-like
Immune-tolerant	Pt.1	100	0	100	74	0	74
	Pt.2	100	0	100	88	0	88
	Pt.3	100	2	98	85	0	85
	Pt.4	98	2	96	73	0	73
	Pt.5	98	0	98	77	0	77
	Pt.6	97	0	97	—	—	—
	Pt.7	91	1	91	71	12	59
HBeAg (+) IA	Pt.8	100	0	100	39	24	15
	Pt.9	100	0	100	83	2	81
	Pt.10	99	2	97	88	0	88
	Pt.11	94	0	94	89	0	89
	Pt.12	86	6	80	79	0	79
	Pt.13	86	0	86	87	1	86
	Pt.14	71	13	58	23	3	20
	Pt.15	58	4	54	50	0	50
	Pt.16	42	4	38	19	2	17
	Pt.17	33	10	24	84	9	75
	Pt.18	33	4	29	21	0	21
	Pt.19	30	0	30	23	1	22
	Pt.20	29	10	20	0	0	0
	Pt.21	14	3	11	56	0	55
	Pt.22	12	1	11	3	1	2
	Pt.23	—	0	0	56	24	32
Inactive chronic hepatitis B	Pt.24	58	78	22	10	12	0
	Pt.25	56	6	90	12	64	0
	Pt.26	41	26	63	2	0	1
	Pt.27	34	0	84	31	0	60
	Pt.28	29	28	54	11	3	2
	Pt.29	17	0	73	7	2	49
	Pt.30	7	9	56	2	1	12
HBeAg (−) IA	Pt.31	100	25	30	12	0	2
	Pt.32	96	0	53	64	2	16
	Pt.33	89	9	44	1	3	0
	Pt.34	84	2	48	60	0	37
	Pt.35	83	6	36	4	0	57
	Pt.36	73	7	34	51	19	21
	Pt.37	65	13	20	12	2	2
	Pt.38	55	12	15	2	5	0
	Pt.39	53	1	23	18	0	10
	Pt.40	53	3	9	3	3	13
	Pt.41	50	12	47	37	8	2
	Pt.42	42	22	34	57	10	2
	Pt.43	40	6	35	40	2	0
	Pt.44	33	14	20	4	31	0
	Pt.45	26	12	18	5	7	4
	Pt.46	24	17	0	10	7	0
	Pt.47	12	3	4	17	1	1

Abbreviations: IA, immune-active; —, not available; Pt., patient.

### Spatial relationship of HBV-infected hepatocytes with pathological features

**FIGURE 2:**
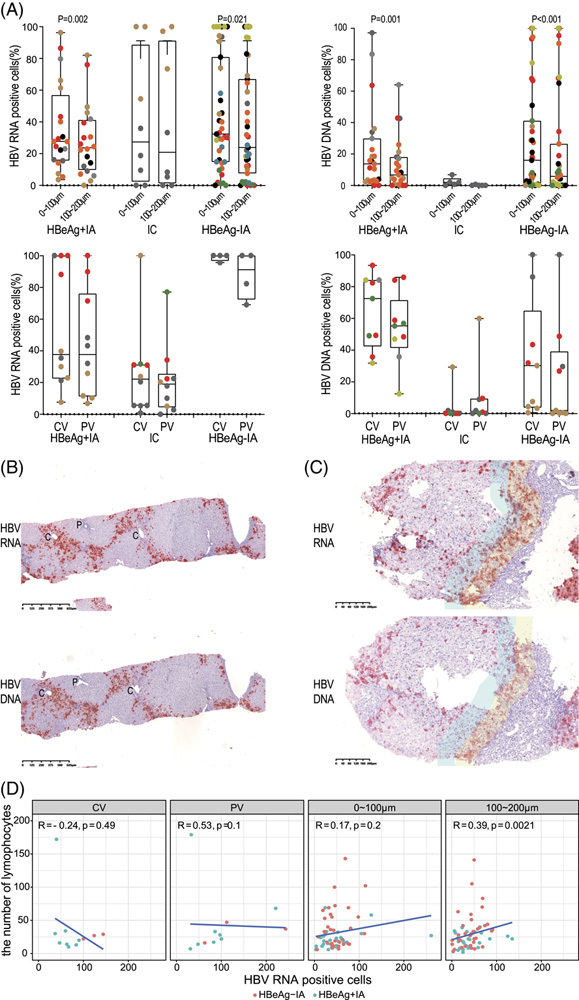
Spatial relationship between HBV-infected hepatocytes and salient pathological features of chronic hepatitis B. (A) Distribution of HBV RNA-positive and DNA-positive hepatocytes in hepatitis B patients. Samples with positivity rates of <80% were included in the data analysis. The percentage of HBV RNA-positive cells from the immune-tolerant (IT) patients are all >80% so that IT patients are not included in this figure. For patients with METAVIR staging scores ≤2, we compared the positive cells around the central venous areas and portal areas. For patients with METAVIR staging scores ≥ 3, we compared the positive hepatocytes that were 0–100 μm and 100–200 μm away from the fibrotic areas. Different symbols represent different patients. The symbols in same color are nonrepetitive fields from 1 patient. (B) One peculiar case wherein the HBV-infected hepatocytes are mostly found in zone 3 of the liver acinus (×40). (C) The yellow areas are located near the fibrotic areas, while the blue areas are 100–200 μm away from the fibrotic area. For HBeAg-positive immune-active (HBeAg+IA) and HBeAg-negative immune-active (HBeAg−IA) patients, more positive hepatocytes were observed near the fibrotic areas (×40). (D) Relationship between lymphocytes and HBV-positive cells in different areas *in situ* in patients with HBeAg+IA and HBeAg−IA. For patients with METAVIR staging scores ≤2, the positive cells and lymphocytes around the central venous areas and portal areas were compared. For patients with METAVIR staging scores ≥3, the positive hepatocytes and lymphocytes that were 0–100 μm and 100–200 μm away from the fibrotic areas were compared. Red points represent patients with HBeAg−IA patients, while blue points represent patients with HBeAg−IA. Abbreviations: CV, central vein; PV, portal vein.

The immune clearance of HBV-infected hepatocytes promotes the distortion of the liver cell plate structure and induces subtle changes in the pathological features of inflammation and fibrosis. For the patients whose liver fibrosis stages was F0, F1, and F2 (METAVIR score), we compared the regions within 100 μm, which contained roughly 5 rows of hepatocytes, around the portal areas and central veins. We found that HBV RNA-positive and DNA-positive cells can be scattered or aggregated in the tissues, regardless of whether they were located close to the portal triad or the central vein (Figure 2A). Notably, in 1 patient in the HBeAg+IA phase, we observed HBV-infected hepatocytes specifically in zone 3 of the functional acinus. This might be a snapshot of the displacement process of infected hepatocytes by proliferating hepatocytes, and that these proliferating hepatocytes are invulnerable to the establishing HBV infection (Figure 2B).

For the patients with more severe fibrosis (stages F3 and F4), we further evaluated the spatial relationship between HBV RNA-positive or DNA-positive hepatocytes and the histological features of fibrosis by comparing the positive cells located within 100 μm around the fibrotic septa and those within the 100–200 μm range. We found that positive cells tended to gather around the fibrotic septa (Figure 2C).

Furthermore, in patients in the HBeAg+IA and HBeAg−IA phases, we analyzed the relationship between necroinflammation and HBV-positive cells in areas close to the portal vein, close to the central vein, or in intralobular hepatic cords (Figure 2D). We found that for patients in the HBeAg+IA phase, the percentage of cells containing cytoplasmic HBV DNA/RNA signals and the inflammation activity (Histological Activity Index score) were negatively correlated, suggesting less liver injury, especially in the phase of IT when viral replication is productive. In contrast, in patients in the HBeAg−IA phase, both the percentage of total HBV RNA-positive cells and the percentage of HBV RNA cytoplasmic positive cells were tightly correlated with necroinflammation, indicating that the viral product is the driving force of immune pathogenesis of chronic hepatitis B in these phases (Supplemental Figure S3, http://links.lww.com/HC9/A229). The relationship between intrahepatic HBV DNA/RNA signals and fibrosis stage was not significant.

### Subcellular patterns of HBV DNA-positive and HBV RNA-positive signals

**FIGURE 3:**
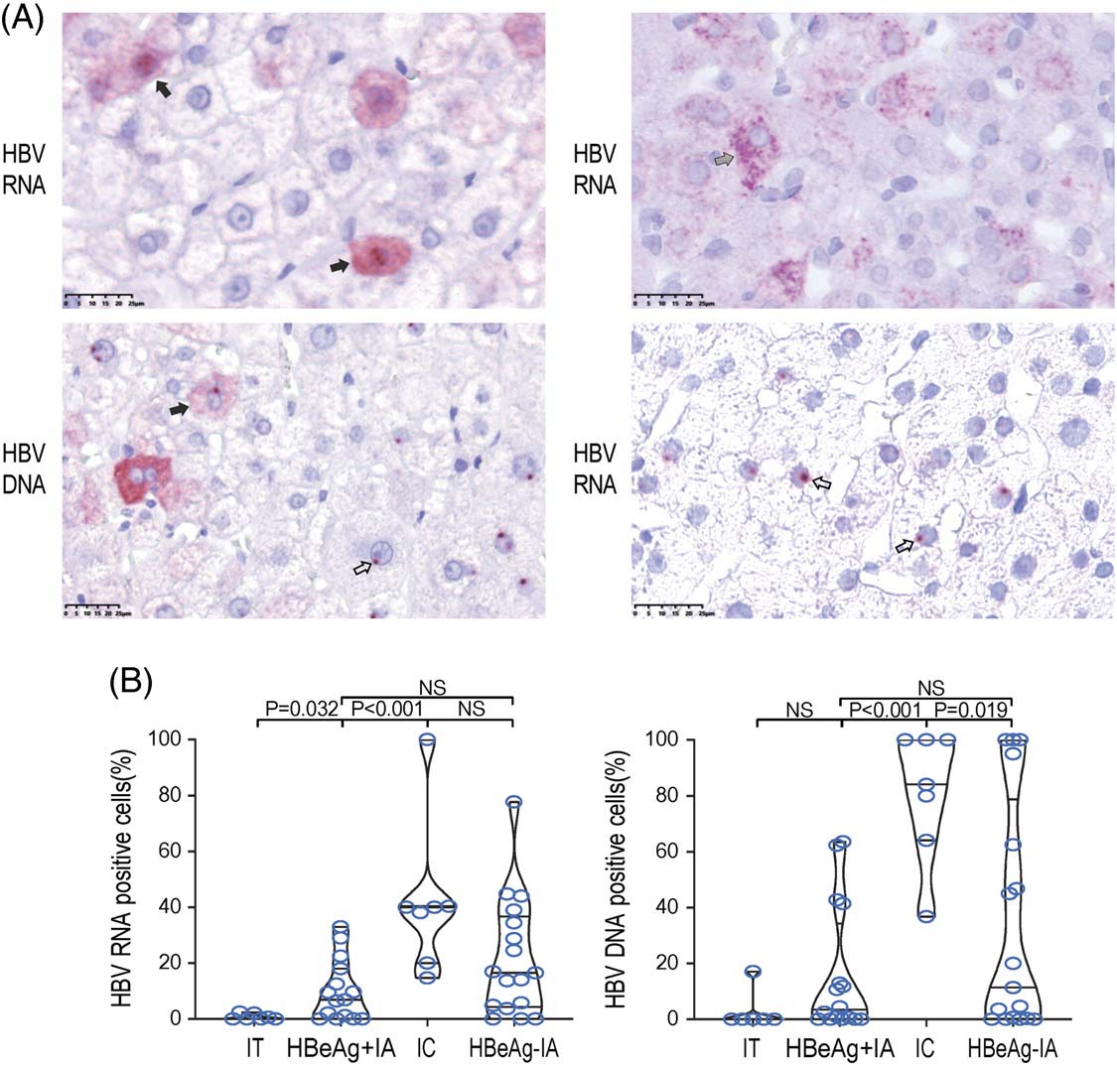
Subcellular localization of positive signals in hepatocytes throughout the natural course of chronic HBV infection. (A) Representative images of hepatocytes that have positive signals in both the cytoplasm and nucleus (solid arrow), and hepatocytes that have positive signals in the nucleus only (hollow arrow). HBV DNA signals appeared to be sand-like in the cytoplasm and punctate in the nucleus, while HBV RNA signals appeared to be sand-like (solid arrow) or punctate (slash arrow) in the cytoplasm and punctate in the nucleus (×400). (B) The percentage of cells whose HBV RNA-positive and DNA-positive signals were only distributed in the nucleus in all kinds of positive cells. Abbreviations: IA, immune-active; IC, inactive chronic hepatitis B; IT, immune-tolerant; NS, nonsignificant.

After browsing >50,000 HBV RNA-positive and DNA-positive hepatocytes, we summarized the subcellular distribution of their signals into several patterns, which correspond to the biogenesis of viral nucleic acids according to the HBV life cycle. Polygonal-shaped cells with well-defined cell borders that are easily visualized owing to the diffuse “sand-like” fine granular signals in the cytoplasm represented hepatocytes with an active viral infection, with HBV nucleocapsid-associated associated pregenomic RNA or relaxed circular DNA. In some infected hepatocytes, HBV RNA signals appeared similar to puncta that are localized to the cytoplasm or close to the nuclear membrane. This distribution could be due to the translation of the HBV integrant mRNA, which utilizes certain cellular machinery. HBV DNA also had similar patterns of subcellular distribution. Signals that are uniquely retained within the nucleus as puncta or appear like several dots are highly indicative of the covalently closed circular DNA (cccDNA) or HBV DNA that may be integrated into the human genome. However, some HBV RNA-positive cells were also identified to have signals that were predominantly confined within the nucleus or annexed to the nucleus, which were reproduced but not understood (Figure 3A). In most hepatocytes, the signals in the cytoplasm were sand-like. Indeed, some areas had cells with both “sand” and “punctum” signals. A few hepatocytes only had focally clustered cytoplasmic punctum signals, which may be due to the clonal expansion of hepatocytes solely containing HBV integrants. In 1 IT patient, almost all signals appeared as puncta, indicating that almost all signals in hepatocytes in the sampling area were from HBV integrants.

**FIGURE 4:**
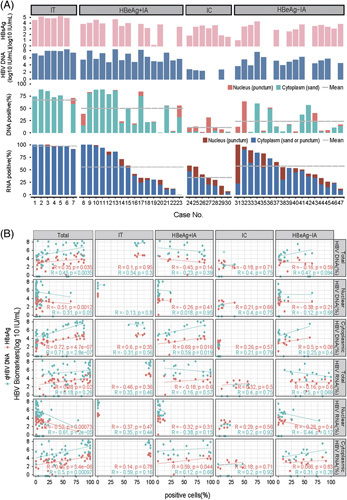
Correlation between HBV-positive signals in the tissue and serum HBV markers. (A) Quantification of HBV-infected hepatocytes and subcellular characteristics of each liver sample from each patient. HBV DNA signals like sand in cytoplasmic and puncture in the nucleus, and, HBV RNA signals like sand or puncture in cytoplasmic and puncture close to the nucleus membrane were incorporated into the analysis. Mean: the average level of positive signals in different chronic hepatitis B stages indicated by a gray dotted line. (B) Correlation between HBV-positive signals *in situ* and serum HBV DNA and HBsAg. For all the patients in natural history, the percentage of cells containing only nuclear HBV DNA signals and the level of serum HBsAg and HBV DNA were negatively correlated. Meanwhile, the percentage of cells containing nuclear and cytoplasmic HBV DNA signals were positively correlated with the level of serum HBsAg and HBV DNA. Total HBV DNA (%): the percentage of HBV DNA-positive cells; total HBV RNA (%): the percentage of HBV RNA-positive cells; nuclear HBV DNA (%): the percentage of HBV DNA-positive signals only in hepatocyte nucleus; cytoplasmic HBV DNA (%): the percentage of HBV DNA-positive signals in hepatocyte cytoplasm; nuclear HBV RNA (%): the percentage of HBV RNA-positive signals only in hepatocyte nucleus; cytoplasmic HBV RNA (%): the percentage of HBV RNA-positive signals in hepatocyte cytoplasm. Abbreviations: IA, immune-active; IC, inactive chronic hepatitis B; IT, immune-tolerant.

16pt?>When comparing all phases, we found that the percentage of cells containing only nuclear HBV DNA signals was significantly higher in the IC phase than in the IT and HBeAg+IA phases (Figures 3B, 4A, Table 2). This indicates that a smaller number of relaxed circular DNA–positive hepatocytes and hepatocytes harbor either transcriptionally silenced cccDNA or HBV integrants that became apparent in the IC phase. The subcellular distribution of HBV RNA also supports this notion, as there is a relatively less active production of nucleocapsid-associated pregenomic RNAs in the IC phase. Collectively, hepatocytes harboring transcriptionally inactive cccDNA or HBV integrants were incapable of undergoing transcription, which became apparent in the IC phase.

### Correlation between intrahepatic HBV DNA-positive and RNA-positive signals and serum HBV DNA load and HBsAg titer

Circulating HBsAg is comprised of cccDNA and integrated HBV DNA origins, whereas HBV replication is only dependent on the cccDNA. We examined the relationship between intrahepatic HBV DNA-positive and RNA-positive signals and the levels of circulating viral biomarkers in clinical use, such as HBsAg and HBV DNA. As expected, the percentage of total HBV DNA-positive hepatocytes was positively correlated with serum HBV DNA load and HBsAg titer, and the percentage of total HBV RNA-positive hepatocytes was positively correlated with serum HBsAg titer. When stratified with the pattern of the subcellular distribution, the percentages of cytoplasmic HBV DNA-positive and RNA-positive hepatocytes exhibited much better correlation with serum HBV DNA load. These results further indicate that the cytoplasmic HBV signals are the source of productive HBV infection. In contrast, nuclear HBV signals and serum viral biomarkers were negatively correlated, which supported the notion that the nuclear HBV signals were less relevant to HBV production (Figure 4B).

## DISCUSSION

The natural history of chronic hepatitis B is crucial in understanding the development of this disease. Observation of liver biopsy specimens through microscopy is a very direct way to visualize viral replication by correlating the morphological changes with disease progression.^5^ *In situ* hybridization has its inherent advantages, including the illustration of critical viral replication intermediates and their spatial relationship to pathobiological changes observed from liver biopsies.^19^ Our study shows that HBV-infected hepatocytes are located nearest to the site of inflammation and fibrous septa, which supports the notion that the immune response against the virus is the driving force for pathological changes.^20^–^24^ However, it still leaves the conundrum of why the productive infection in IT subjects does not lead to immunopathological injury. Our findings also provoke us to question whether viral replication between HBeAg-positive and HBeAg-negative immune clearance has a clear difference, given that they are not distinguished by either the percentage of HBV-infected cells or subcellular distribution of signals. HBe seroconversion might have exclusively stemmed from host immunological factors rather than viral replication.

Pieces of evidence in this study do not support the vulnerability of human hepatocytes to HBV infection is influenced by oxygen, and nutrient metabolism and detoxification, especially since infected cells and the functional zonation of the liver do not have an apparent spatial relationship.^25^–^27^ First, almost every hepatocyte in the IT phase was infected. Second, infected hepatocytes in the later phases are scattered across the lobule. These suggest that no matter where the hepatocyte is located, it can support and maintain the HBV infection. Interestingly, in 1 patient, we observed that HBV-infected hepatocytes were specifically located in zone 3 of the functional acinus, whereas hepatocytes in zones 1 and 2 are free from infection. From the structure of the acinus, the most likely interpretation is that the hepatocytes in zones 1 and 2 are proliferating, which makes them invulnerable to an establishing HBV infection; this is similar to our observation in HCC, where the proliferating hepatocytes were negative for HBV, as examined using the *in situ* hybridization approach (data not shown).^28^,^29^

The persistence of cccDNA is a hurdle to the cure of chronic hepatitis B. In addition, HBV integration is also an important source of HBsAg, making it difficult to achieve a functional cure.^30^–^33^ Our observation once again confirms this finding. The circulating HBsAg comprises cccDNA and integrated HBV DNA origins, wherein only the cccDNA origin is viral replication-dependent. We examined whether HBsAg adjusted by the number of cytoplasmic positive cell show a better correlation with HBV DNA load. The results showed that the concentration of adjusted HBsAg was better correlated with HBV DNA load. Furthermore, we have observed cells with exclusive HBV DNA nuclear signals and cytoplasmic punctum signals of HBV RNA in every phase of the natural HBV history. These kinds of signals may be derived from integrated HBV. Recent studies have found that HBV integration occurs at every stage, even in the IT phase.^12^,^34^ The IC phase has the highest percentage of hepatocytes with the HBV integrants,^35^ which indicates that HBV-integrated cells escape immune clearance and continue to expand when the hepatocytes proliferate. Some studies reported many HBV-integrated pieces and suggested that HBV-integrated hepatocytes may lead to the extensive expansion of cells and eventually cause carcinoma.^36^–^39^ In most cases, we did not observe a large area of such cells around the portal area or along the liver cell plate; these cells were often distributed as small clusters or patches, whereas clonal expansion was observed in some fields. The results of *in situ* hybridization suggest that the expansion of HBV-integrated hepatocytes may be the result of expansion induced by liver homeostasis or may indicate preconditions of dysplasia.^28^

In this study, we developed probes for the plus and minus strands of HBV to visualize its transcription products as well as the relaxed circular DNA in the cytoplasm and to distinguish hepatocytes that harbor cccDNA or HBV integrants. Hepatocytes with productive HBV infection could be defined as being positive for both cytoplasmic HBV RNA and HBV DNA, which can easily be seen due to their polygonal pattern. Hepatocytes harboring HBV integrants could be defined as having a single nuclear dot when hybridized with the HBV DNA probe and several cytoplasmic puncta when hybridized with the HBV RNA probe. Hepatocytes harboring transcriptionally inactive cccDNA or HBV integrants could be defined as having a single nuclear dot when hybridized with an HBV DNA probe and no signal when hybridized with an HBV RNA probe (Graphical abstract).

Constrained by the limited access to liver biopsy tissues of untreated patients and their geographic locations, we only included 47 patients diagnosed with chronic HBV genotypes B and C infections. Although we did not perform molecular biological verification, our interpretation of the characteristics of HBV signal distribution in the liver is consistent with the findings of current studies and the characteristics in the liver of patients after antiviral therapy.^40^

Therefore, we advocate including *in situ* hybridization in routine histological evaluation of chronic hepatitis B, which offers substantial information on viral replication. Our findings helped us understand the viral-host interaction and the relationship between anatomical structures and disease stages across the natural course of chronic HBV infection.

## Supplementary Material
