# Supplementary figures and images for: Visualizing in situ viral replication across the natural history of chronic HBV infection
Source: Hepatol Commun. 2023 Mar 30;7(4):e0111. doi: 10.1097/HC9.0000000000000111 (PMC10069836; doi:10.1097/HC9.0000000000000111)

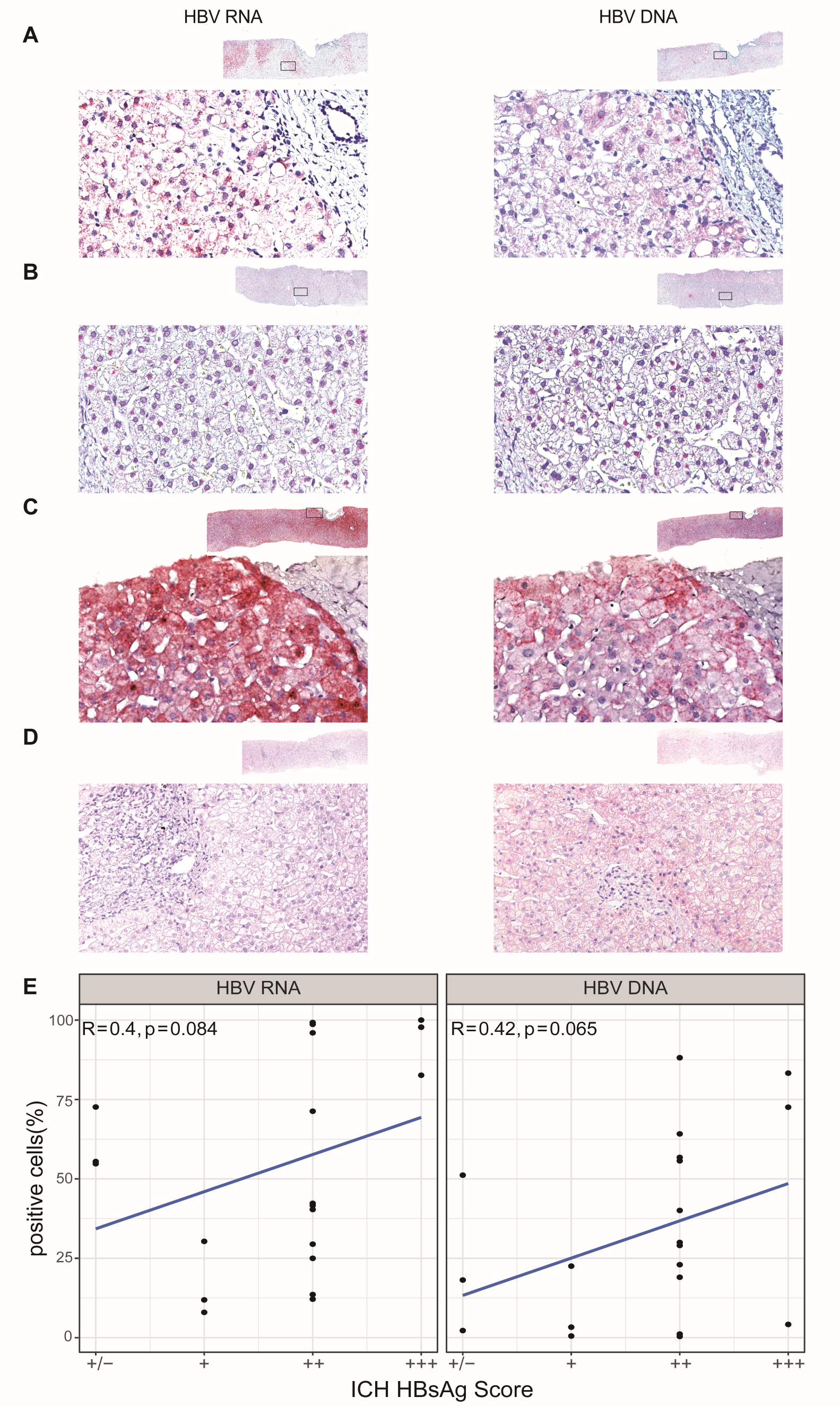

Supplement: Supplementary file 1 [file hc9-7-e0111-s001.tif]

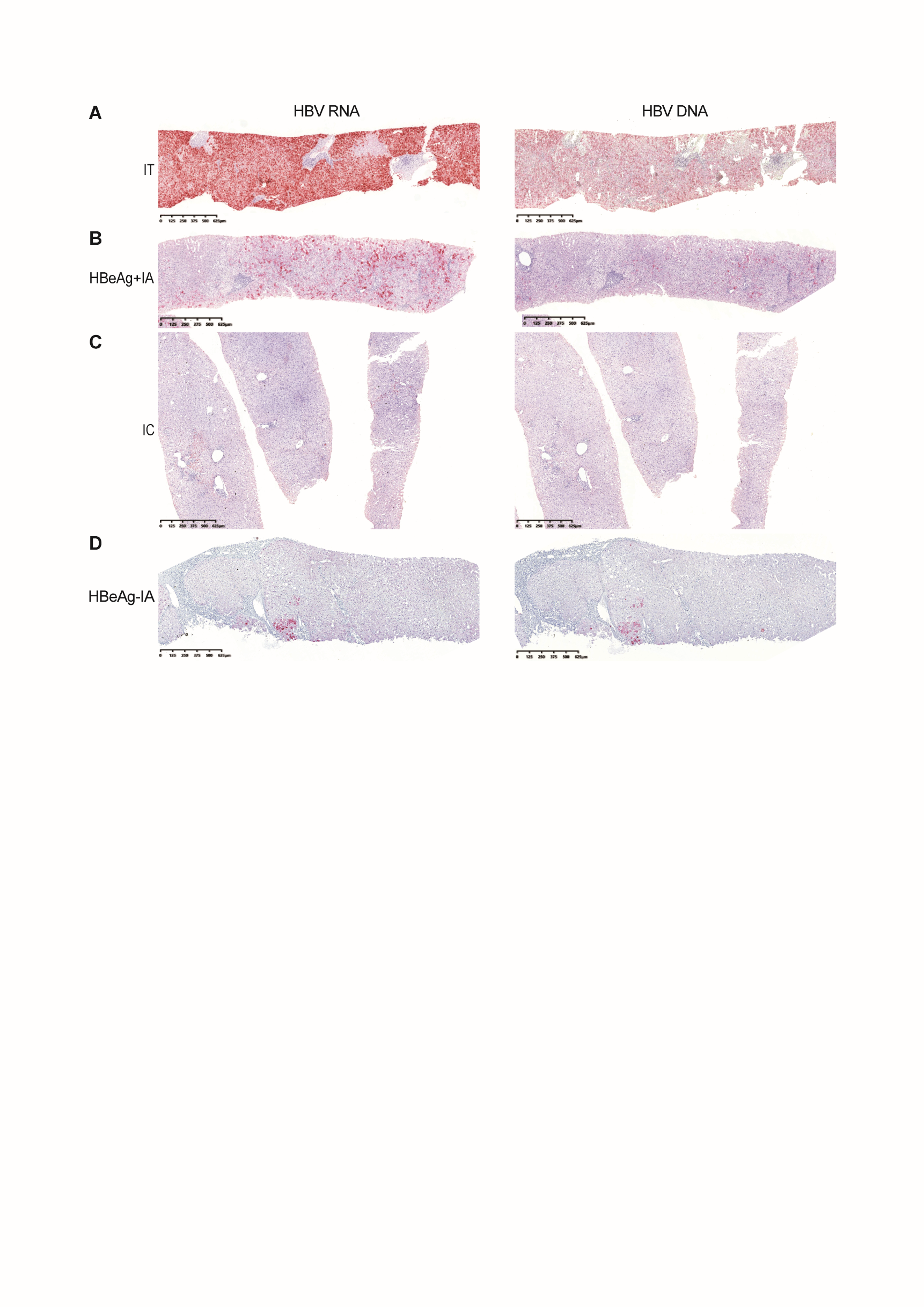

Supplement: Supplementary file 2 [file hc9-7-e0111-s002.tif]

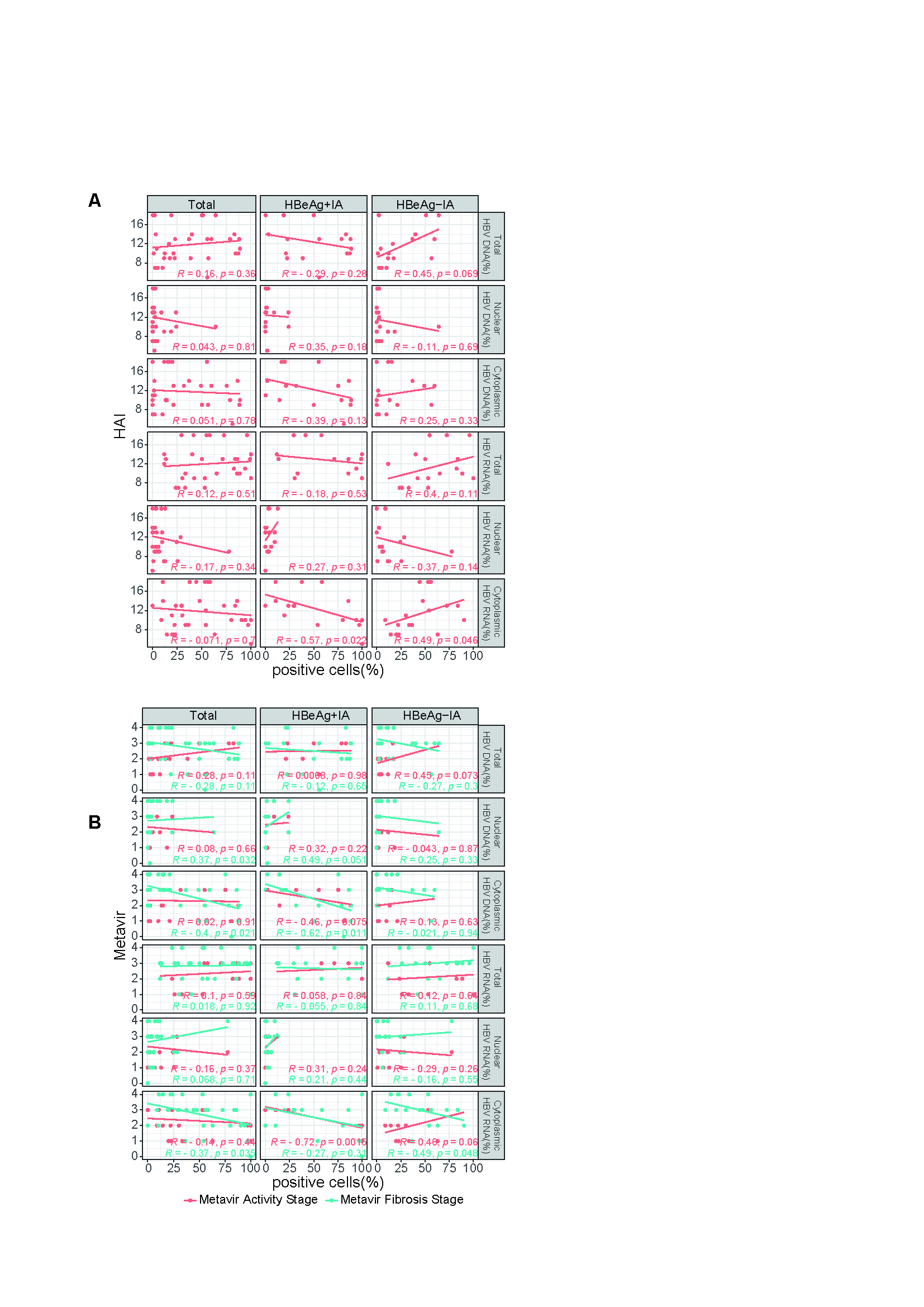

Supplement: Supplementary file 3 [file hc9-7-e0111-s003.tif]
